# Human Functioning: Developments and Grand Challenges

**DOI:** 10.3389/fresc.2020.617782

**Published:** 2021-03-10

**Authors:** Jerome Bickenbach

**Affiliations:** ^1^Swiss Paraplegic Research, Nottwil, Switzerland; ^2^Department of Health Sciences and Medicine, University of Lucerne, Lucerne, Switzerland

**Keywords:** functioning, challenge, epidemiology, clinical practice, research, International Classification of Functioning, Disability and Health

## ICF and Functioning: the Paradigm Shift

Like many ground-breaking innovations, the conceptual thinking that went into The World Health Organization's *International Classification of Functioning, Disability and Health* (ICF) in the early 1990s had been anticipated in the literature for decades, although formally endorsed by the WHO in 2001, the result was a true paradigm shift (1). With the ICF, WHO made it clear that, although health states are purely biological phenomena, what matters to people about their health is not merely these biological processes but also the concrete impact on their daily lives: what they can do and be, the actions they perform and the life goals and aspirations they can achieve. The ICF made it clear that challenges to our health—disease, injuries, and the natural process of aging itself—bring about decrements in body functions and alterations of body structure, and these changes, in interaction with the environment, can negatively affect—sometimes trivially, sometimes profoundly—what we can do and achieve in our lives.

To capture this multifaceted and continuous phenomena, the ICF proposes the term **functioning**—the sum total of functions and structures of the body and mind, the actions people perform, and the complex and socially-embed life activities they participate in. Functioning, as a term of science, requires both a conceptual description or model and, for scientific description, operationalization and measurement, a classification of the lived experience of health. The ICF provided both. The notion of functioning has in the last 20 years made it possible to clarify the concept and practice of healthcare, and most particular the concept and practice of rehabilitation. The ICF notion of functioning provides a clearer understanding of the health and social impact of future trends in population aging and increased prevalence of non-communicable diseases, trends that will reveal the increasing need for, and social value of rehabilitation as a health strategy.

Since 2001, the ICF has been widely and diversely applied as a standard classification, an **international reference language** for the collection of information about the lived experience of health. The ICF complements and supplements the WHO's *International Classification of Diseases* (ICD) (2) as well as, more recently, the *International Classification of Health Intervention* (ICHI) (3). WHO's primary purpose in promulgating each of these standards is to ensure comparability of international health information—information that is of practical use to practitioners and researchers to explain and influence functioning both clinically and at the population level, and to policy-makers striving to improve the performance of national health systems to respond to the functioning needs of individuals and populations. The future of e-health and all digital applications of health information depends on data standardization, as does a more comprehensive epidemiology that goes beyond the standard health indicators of mortality and morbidity.

The conceptual foundations of the ICF notion of human functioning has also spurred research and applications that have had a fundamental and diverse impact on health sciences and health and social policy. Functioning is conceptualizes in the ICF in terms of a person-environment interaction, which in turn has led to the important conceptual distinction between a person's intrinsic health **capacity** and the person's actual, real world **performance** in which her or his physical, human-built, attitudinal, and socio-political environment may hinder or enhance performance. This model has been particularly useful in clarifying the notion of disability as a problem, decline, or non-optimal functioning in one or more domains. This understanding of disability has lead to a rethinking of the most prominent policy applications of the notion of disability, and in particularly that of disability assessment and determination processes for health and social benefits, including the need for vocational rehabilitation. Rather than understanding disability purely from the perspective of biomedical phenomena, the ICF has underwritten the more robust and valid notion of disability as the outcome of an interaction between intrinsic health capacity, personal factors, and the environment.

The last two decades of research and application of the ICF and its key notion of functioning point to an active future of grand challenges for rehabilitation.

## Clarification of the Aim and Scope of Rehabilitation as a Health Strategy

As one of the five health strategies recognized by the WHO, rehabilitation has historically been undervalued and misunderstood, in part because, unlike curative medicine and health promotion and disease prevention, rehabilitation seemed to have a somewhat vague aim and purely reactive posture. Recent work on the conceptualization of rehabilitation (4–7), however, has argued that the notion of functioning may be the key to a new understanding in which the aim of rehabilitation is to optimize functioning in the face of demographic and epidemiological trends that point to a future of increased population disability. It remains a challenge how this insight can be used to further clarify the role and purpose of rehabilitation as a fundamental health strategy. Equally challenging is to ensure that rehabilitation is not merely a high-income country health strategy but its aim and scope can be effectively implemented in low- and middle-income countries as well.

## Rehabilitation assessment and Evaluation Using Functioning

In practical terms, clinicians require tools and an operational language in which to assess their patients and evaluate the quality and effectiveness of rehabilitation interventions. Given the importance of this area of clinical practice, there is a growing literature on the use of ICF and functioning in the development of clinical assessment, evaluation and quality management tools and methodologies (8–11). The challenge in the future is to both to continue this development, and as far as possible, to ensure comparability between approaches to rehabilitation assessment and evaluation, and recognition of the importance of rehabilitation across health and social sectors.

## Functioning and Rehabilitation Goal-Setting and Outcome Measures and Tools

The other major component of intervention planning for rehabilitation is goal-setting that is patient-oriented (12–14) as well as the development and application of clinical (15–17) and patient-reported (18, 19) outcome measures and related tools that reflect these goals. There is perhaps nothing more important to rehabilitation as a clinical practice than that practitioners are able to set goals and identify outcomes that represents what is of importance to the beneficiary of rehabilitation interventions. For this the notion of functioning is ideal as it captures the lived experience of health and so what actually matters to be able their health in the daily lives and over the life course.

## ICF as Reference Framework for Functioning Information

For functioning to play a role in clinical practice—either in assessment, goal-planning, outcomes, and evaluation—work needs to be done on standardized clinical reporting of the functioning information (20, 21). The case needs to be made, to the satisfaction of health policy-makers, that functioning information should be fully integrated into health information systems (22, 23), potentially within electronic health records (24). Although the groundwork for using ICF as an international reference framework for functioning information has been laid, the challenge remains to break down the barriers to an increased demand for functioning information from clinical and a recognition on the part of policy makers of the need for routine collection of functioning information in order to strengthening rehabilitation within the health system. A companion challenge is to facilitate the role of functioning information in evidence-based policy development, responsive to the relevance and value of this information, not merely in the health sector, but in the social, labor, and education sectors as well.

## Economic Evaluation of Rehabilitation Interventions

One of the challenges identified in the WHO?s call for action **Rehabilitation 2030** is to convince countries of the importance of strengthening rehabilitation within national health systems, especially in light of anticipated increased need for rehabilitation given population aging and the increased prevalence of non-communicable diseases (25). Making this case, at the national level, requires a demonstration that rehabilitation is not an added cost, but an important economic investment. The economic argument, recent work has suggested, can best be made where the economic benefit of rehabilitation is expressed in terms of functioning improvements (26, 27), but far more work needs to be done in order to fully make the economic investment argument.

## Work Capacity Assessment for Vocational Rehabilitation

Around the globe, countries are realizing that it no longer makes economic or political sense to use pensions and other social policy mechanisms to exclude people experiencing disability from entering and participating in the labor market. With support, even those with severe and long-lasting impairments can be employed: it is a fundamental human rights issue (28). Vocational rehabilitation can open the door to employment and the notion of functioning has been shown to be the key metric for assessment of work capacity (29, 30), the need for vocational rehabilitation (31), and job matching (32). It remains a challenge to explore the role of functioning as the basis for return-to-work and other work related social strategies, and developing tools that ensure that people experiencing disability are, and remain, active participants in the labor market.

## Development of Environmental Measurement Tools

At the heart of the ICF concept of functioning is the insight that human functioning is an outcome of complex interactions between a person's intrinsic health capacity, determined by the extent and severity of health conditions that are present, and the physical, human-being, attitudinal, social, and political environment in which the individual lives and carries out her or his life. This constituents ICF's “paradigm shift” in the understanding of the concepts of functioning, disability, and health. Yet, although the health sciences have made considerable progress toward understanding and explaining the kinds of problems and limitations in health capacity that human experience, we are just beginning to develop the science of describing and measuring the impact of the environment on functioning (33, 34). Understanding how our environment, in all of its diversity, shapes human functioning remains a challenge for the future.

## Prospects of an Epidemiology of Functioning

Finally, and in a sense most fundamentally, the notion of functioning has the potential of reorienting the basic health science of epidemiology itself, an epidemiology that includes but moves beyond biomedical epidemiology toward a more comprehensive understanding of health: an epidemiology of functioning in the light of health conditions (35). An epidemiology of functioning would seek to understand the health capacity and environmental determinants of people's actual experience of living with health conditions in their environments. Developing population metrics of functioning (36) and with the availability of very large data sets constructing functioning trajectories of aging (37) should assist in identifying identify epidemiological patterns that would greatly enhance our ability to determine the efficacy of functioning-based intervention along the life-course. As our understanding of functioning improves, and our assessment and measurement instrumentation is perfected, the challenge of a true epidemiological of functioning may be within our reach.

## Conclusions

The notion of human functioning, grounded in the ICF has in the past two decades led to a growing body of scientific literature in health generally, and rehabilitation in particular. These challenges, and undoubtedly many others that reveal themselves in future years, will raise questions that demand the highest quality scientific investigation and research. The future for research in human functioning is indeed bright.

## Author Contributions

The author confirms being the sole contributor of this work and has approved it for publication.

## Conflict of Interest

The author declares that the research was conducted in the absence of any commercial or financial relationships that could be construed as a potential conflict of interest.
